# Downregulation of the *Drosophila* Immune Response by Peptidoglycan-Recognition Proteins SC1 and SC2

**DOI:** 10.1371/journal.ppat.0020014

**Published:** 2006-02-24

**Authors:** Vincent Bischoff, Cécile Vignal, Bernard Duvic, Ivo G Boneca, Jules A Hoffmann, Julien Royet

**Affiliations:** 1 Institut de Biologie Moléculaire et Cellulaire, UPR 9022 du CNRS, Strasbourg, France; 2 Unité EMIP UMR INRA-UMII 1133, Université Montpellier II, Place Eugène Bataillon, Montpellier, France; 3 Unité de Pathogénie Bactérienne des Muqueuses, Institut Pasteur, Paris, France; 4 IBDM/LGPD, Campus de Luminy, Marseille, France; Stanford University, United States of America

## Abstract

Peptidoglycan-recognition proteins (PGRPs) are evolutionarily conserved molecules that are structurally related to bacterial amidases. Several *Drosophila* PGRPs have lost this enzymatic activity and serve as microbe sensors through peptidoglycan recognition. Other PGRP family members, such as *Drosophila* PGRP-SC1 or mammalian PGRP-L, have conserved the amidase function and are able to cleave peptidoglycan in vitro. However, the contribution of these amidase PGRPs to host defense in vivo has remained elusive so far. Using an RNA-interference approach, we addressed the function of two PGRPs with amidase activity in the *Drosophila* immune response. We observed that PGRP-SC1/2–depleted flies present a specific over-activation of the IMD (immune deficiency) signaling pathway after bacterial challenge. Our data suggest that these proteins act in the larval gut to prevent activation of this pathway following bacterial ingestion. We further show that a strict control of IMD-pathway activation is essential to prevent bacteria-induced developmental defects and larval death.

## Synopsis

It has long been known that the mammalian immune response needs to be kept under tight control. Responses that are delayed or of insufficient vigor can lead to a failure to control infection. However, excessive or inappropriate inflammation can be harmful or event fatal. Using the fruit fly as a model, evidence is presented that such an immuno-modulation is also essential in invertebrates and is mediated by peptidoglycan-recognition proteins (PGRPs). PGRPs are evolutionarily conserved molecules derived from enzymes that cleave bacterial peptidoglycan. It has been shown previously that some PGRPs have lost this enzymatic activity and function as sensors of bacteria upstream of the *Drosophila* immune pathways. The contribution of PGRPs which have maintained enzymatic activity to host defense has remained elusive so far. Here, the authors investigate in vivo data on the role of *Drosophila* PGRPs with enzymatic activity. Their results suggest that these proteins are required in the larval gut to negatively regulate the immune response, thus preventing bacterially induced developmental defects and death.

## Introduction

The antimicrobial host defense of *Drosophila* involves rapid synthesis of small-sized cationic peptides by the fat body [1,2]. These antimicrobial peptides are released into the open circulatory system where they attack invading microorganisms. The transcription of the genes encoding these peptides is under the control of two distinct signaling pathways. The Toll pathway, which is primarily activated after gram-positive bacterial and fungal infections, controls the expression of drosomycin, an antifungal peptide, together with many other genes via the NF-κB–family member DIF (dorsal-related immune factor) [3,4]. The second cascade, known as the IMD (immune deficiency) signaling pathway, is predominantly triggered after gram-negative infection and regulates, via the NF-κB protein Relish, the synthesis of some antibacterial peptides and many other genes [5,6]. An efficient Toll-pathway activation after gram-positive bacterial infection requires the function of at least three soluble proteins, namely peptidoglycan-recognition protein-SA (PGRP-SA) [7], PGRP-SD [8], and gram-negative binding protein-1 [9,10]. On the other hand, sensing of gram-negative bacterial infection has been shown to be dependent on two other PGRP family members, PGRP-LC [11–13] and PGRP-LE [14,15].

The ability of *Drosophila* to discriminate between gram-positive and gram-negative bacteria relies on the specific recognition of different forms of peptidoglycan (PGN) [16,17]. Bacterial PGN consists of long carbohydrate chains of alternating *N*-acetylglucosamine and *N*-acetylmuramic acid connected via stem peptides [18] (Figure S1). Most PGNs from gram-positive bacteria contain l-lysine in the third position of the stem peptide (Lys-PGN) and are recognized by PGRP-SA and PGRP-SD [7–9]. In PGN of gram-negative bacteria and in that of gram-positive bacilli, the lysine residue is replaced by meso-diaminopimelic acid (m-DAP) (Figure S1). This second type of PGN (m-DAP-PGN) is sensed by PGRP-LC and PGRP-LE receptors, leading to the activation of the IMD pathway [14–17,19].

PGRPs form a large group of proteins present in insects and mammals [20–25], which have in common a 160-amino acid–domain with striking sequence similarity to *N*-acetylmuramyl-l-alanine amidases (NAMLAA) [26]. These bacterial enzymes hydrolyze the bond formed between the lactyl group in *N*-acetylmuramic acid and the l-alanine in the stem peptide of PGN. In some of these PGRP molecules, the amidase function is conserved, as documented for *Drosophila* PGRP-SC1 [27] and PGRP-LB [28], and for mouse and human PGRP-L [29,30]. In others, such as in PGRP-SA, SD, LE, or LC, the replacement of a critical cysteine residue within the PGRP domain abolishes this enzymatic function [27]. On the basis of genetic experiments, it is assumed that PGRPs without amidase activity serve as recognition receptors for microbial PGN. However, the in vivo function of PGRPs with amidase activity remains unclear, and *PGRP-L* mutant mice show no immune phenotype. On the basis of in vitro experiments, it has been proposed that amidase PGRPs could act as scavenging molecules. Indeed, degradation of PGN by *Drosophila* PGRP-SC1b markedly reduces its immuno-stimulatory potency in cell-culture assays [27]. We report here an in vivo study on the role of *Drosophila* PGRPs with amidase activity. We show that two PGRPs with described amidase activity, namely PGRP-SC1 and PGRP-SC2, control the intensity of the *Drosophila* immune response. We also present evidence that in the absence of such a control, infection-induced IMD-pathway over-activation can cause developmental defects and larval death.

## Results

### Loss-of-Function Mutants for PGRP-SC1/2 Are Generated by RNA Interference

**Figure 1:**
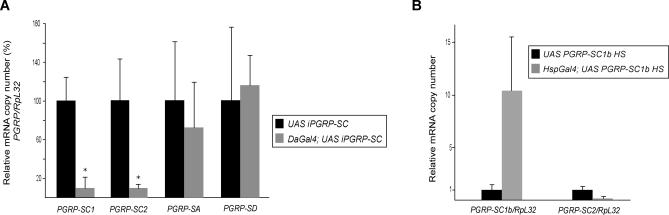
Specific Reduction of *PGRP-SC1/2* mRNA Using RNA Interference In Vivo Each histogram corresponds to the mean value of four independent experiments (± standard deviation). (A) mRNA-level quantification for different PGRPs *(PGRP/RpL32)* shows that *PGRP-SC1* and *PGRP-SC2* mRNA levels are severely reduced in *DaGal4;UAS iPGRP-SC* flies as compared to *UAS iPGRP-SC* control flies. *PGRP-SA* and *PGRP-SD* mRNA levels are not affected. One hundred percent corresponds to the wild-type value for each transcript. Asterisks indicate that the difference between *UAS iPGRP-SC* and *DaGal4;UAS iPGRP-SC* values is statistically significant (*p* < 0.05). (B) Primer specificity in RT-PCR experiments shown by quantification of *PGRP-SC1b* and *PGRP-SC2* transcripts. *PGRP-SC1b* over-expression in *HspGal4;UAS PGRP-SC1b* flies 1 h after a 30-min heat-shock (37 °C) treatment (HS) is well detected with *PGRP-SC1* primers but not with those for *PGRP-SC2.* We can infer that the *PGRP-SC2* primers used in this study are able to discriminate between *PGRP-SC2* and *PGRP-SC1b* transcripts.

In order to address the function of NAMLAA PGRPs under in vivo conditions, we analyzed the immune response of *Drosophila* with reduced PGRP-SC1 and PGRP-SC2 levels. The *Drosophila* genome contains a cluster of two tandemly arranged *PGRP-SC1* loci (named *a* and *b*) and a single *PGRP-SC2* locus [25] (Figure S2A). The two *PGRP-SC1* mRNAs differ by only three nucleotides and translate into a unique protein that is 70% identical to the PGRP-SC2 polypeptide (Figure S2B and S2C). Furthermore, PGRP-SC1 and PGRP-SC2 proteins form a separate cluster in the PGRP phylogenetic tree (Figure S2D). To eliminate potential problems of functional redundancy between these two homologous enzymes, we decided to simultaneously knockdown the *PGRP-SC1* and the *PGRP-SC2* genes in vivo. The presence of long stretches of identical sequences in their transcripts prompted us to take advantage of the RNA-interference method (Figure S2B). Using *PGRP-SC1*– and *PGRP-*SC2–specific primers*,* we could demonstrate that adult flies carrying a *UAS iPGRP-SC* construct (see Materials and Methods) together with a ubiquitous *Gal4* driver *(DaGal4)* exhibited a 90% reduction of both *PGRP-SC1* and *PGRP-SC2* mRNA levels (Figure 1). The transcript levels of *PGRP-SA* and *PGRP-SD,* two closely related family members, were unaffected in these flies, demonstrating the specificity of the designed *UAS iPGRP-SC* construct (Figure 1).

### IMD Pathway in PGRP-SC1/2–Depleted Flies Is Over-Activated

**Figure 2:**
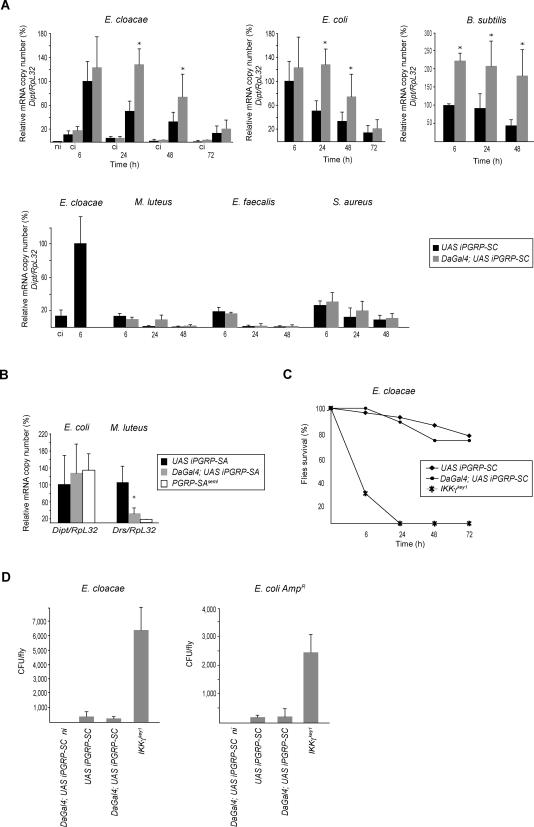
IMD-Pathway Activation Is Downregulated by PGRP-SC1/2 (A) Kinetics of *diptericin* mrna induction *(Dipt/RpL32)* after infection by various bacteria. Each histogram corresponds to the mean value of five independent experiments (± standard deviation). Asterisks indicate that the difference between *DaGal4;UAS iPGRP-SC* and control *UAS iPGRP-SC* values is statistically significant (*p <* 0.05). One hundred percent corresponds to the level of activation at 6 h in control flies. In the lower panel, *diptericin* induction after gram-positive bacterial infections were compared to that obtained after infection by E. cloacae (100%). *RpL32* is used as an internal control. ci, clean injury; ni, noninfected. (B) Quantification of *diptericin* mRNA levels in *UAS iPGRP-SA*, *DaGal4;UAS iPGRP-SA* and *PGRP-SA^seml^* flies shows that reduction of *PGRP-SA* mRNA levels does not influence IMD-pathway induction 6 h after infection by *E. coli.* Quantification of *drosomycin* mRNA levels 24 h after M. luteus infection indicates that PGRP-SA is efficiently knocked down by dsRNA interference. *PGRP-SA^seml^* is a complete loss-of-function mutant for PGRP-SA. Each histogram corresponds to the mean value of five independent experiments (± standard deviation). Asterisk indicates that the difference between *DaGal4;UAS iPGRP-SA* and control *UAS iPGRP-SA* values is statistically significant (*p* < 0.05). (C) *DaGal4;UAS iPGRP-SC* flies are as susceptible to infection by E. cloacae as control flies*.* (D) E. cloacae and *E. coli AmpR* growth in various genetic backgrounds 24 h after infection. Flies with reduced levels of PGRP-SC1/2, unlike *IKK*γ^key1^ mutants, are able to clear bacteria from their hemolymph. Each histogram corresponds to the mean value of four independent experiments (± standard deviation).

We first analyzed the potential role of PGRP-SC in adult flies, which are more amenable than larvae to pricking and survival experiments. For this, we infected *UAS iPGRP-SC* and *DaGal4;UAS iPGRP-SC* flies (*DaGal4* is a ubiquitous driver) with gram-negative bacteria and measured *diptericin* transcript levels as a conventional readout for IMD-pathway activation. Six hours after infection with Enterobacter cloacae or Escherichia coli (a time-point that corresponds to the peak of *diptericin* mRNA kinetics in wild-type flies), no differences were noted between the levels of *diptericin* mRNA in *UAS iPGRP-SC* and *DaGal4;UAS iPGRP-SC* flies (Figure 2A). However, whereas the *diptericin* mRNA level dropped significantly at 24 and 48 h in *UAS iPGRP-SC* flies (as it usually does in the wild-type condition), it remained high in *DaGal4;UAS iPGRP-SC* flies. Similar results were obtained with another m-DAP-PGN–containing bacteria *(Bacillus subtilis),* although the differences between *UAS iPGRP-SC* and *DaGal4;UAS iPGRP-SC* could be detected as soon as 6 h after infection (Figure 2A). Experiments performed with another ubiquitous *Gal4* driver *(ActinGal4)* and an independent *UAS iPGRP-SC* insertion generated identical results (data not shown). When gram-positive bacteria were used as inducers, two of them *(Micrococcus luteus* and *Enterococcus faecalis)* did not activate the IMD pathway above clean injury levels. A third one, *Staphylococcus aureus,* triggered slightly higher *diptericin-*transcription levels in PGRP-SC–depleted flies than in controls, although the induction was mild, as expected from gram-positive bacteria (Figure 2A)**.**

These results indicate that depletion of PGRP-SC induces an over-activation of the IMD pathway after bacterial infection. This phenotype was not observed in noninfected *DaGal4;UAS iPGRP-SC* flies or after pricking with a clean needle (Figure 2A). Altogether, this indicates that the observed effects are dependent on the presence of bacteria and do not correspond to a constitutive activation of the IMD pathway in PGRP-SC–depleted flies. They also demonstrate that the other putative secreted *Drosophila* amidases (PGRP-SB1 and PGRP-SB2) are not able to compensate for the absence of PGRP-SCs in vivo. In the absence of loss-of-function mutants for each of the three *PGRP-SC* genes, we can obviously not rule out the possibility that only one or two of them are responsible for the observed phenotype. To obtain additional proof that the effects obtained in PGRP-SC–depleted flies were specific, we performed similar experiments in flies in which the non-amidase bacterial receptor PGRP-SA had been depleted by RNA interference. Whereas *DaGal4;UAS iPGRP-SA* flies showed an expected reduced ability to respond to infection by the gram-positive *M. luteus,* their response to E. coli remained wild-type (Figure 2B)*.* Therefore, *DaGal4;UAS iPGRP-SA* flies behave as classical *PGRP-SA^seml^* loss-of-function mutants [7] and not like flies with reduced PGRP-SC levels.

### PGPR-SC1/2–Depleted Flies Are Able to Clear Bacteria

Since PGRP-SC1 has been proposed to act as a scavenger molecule [27], we tested whether the IMD-pathway over-activation observed in PGRP-SC–depleted flies could reflect the inability of these flies to clear bacteria. If this were to be proved the case, accumulation of bacteria in the body cavity of *DaGal4;UAS iPGRP-SC* flies could explain the over-activation of the IMD pathway. To test this hypothesis, we compared bacterial loads and survival curves of *IKKγ^key1^, UAS iPGRP-SC,* and *DaGal4;UAS iPGRP-SC* flies infected with *E. cloacae* or with E. coli (Figure 2C and 2D). Whereas *IKKγ^key1^* mutant flies showed a very high bacterial load and a strong susceptibility to these bacteria, no such phenotypes were observed in *DaGal4;UAS iPGRP-SC* flies. Similar results were obtained with B. subtilis (unpublished data)*.* This suggests that the over-response observed in PGRP-SC–depleted flies did not result from an uncontrolled bacterial growth in the hemolymph, and that the role of PGRP-SC proteins in vivo is not to scavenge bacteria from the circulating hemolymph.

### Toll-Pathway Activation Is Wild-Type in *PGRP-SC1/2* Mutant Flies

**Figure 3:**
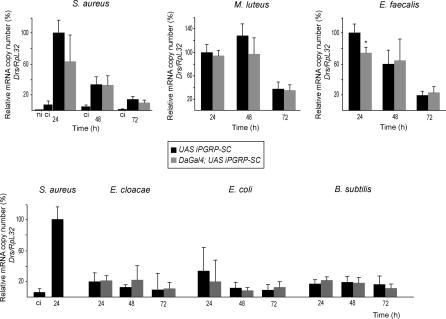
Toll-Pathway Activation Is Wild-Type in *DaGal4;UAS iPGRP-SC* Flies Kinetics of *drosomycin* mrna induction *(Drs/RpL32)* after infection by gram-positive (upper panel) and gram-negative (lower panel) bacteria. Each histogram corresponds to the mean value of six independent experiments (± standard deviation). Asterisk indicates that the difference between *DaGal4;UAS iPGRP-SC* and control *UAS iPGRP-SC* values is statistically significant (*p <* 0.05). One hundred percent corresponds to the level of activation 24 h after infection in control flies. In the lower panel, *drosomycin* induction after gram-negative bacterial infections is compared to that of S. aureus infection which is set to 100%. *RpL32* is used as an internal control.

It is notable that non-enzymatic PGRPs, such as PGRP-SA, PGRP-LC, or PGRP-LE are able to discriminate between Lys-type and m-DAP-type PGN [14–17] (Figure S1). Recent experiments have nevertheless demonstrated that PGRP-SC1b can act as a cleaving enzyme for both gram-positive and gram-negative bacterial PGN in vitro. We therefore asked whether reducing the endogenous levels of PGRP-SC1/2 could also have an effect on Toll-pathway activation by gram-positive bacteria. As illustrated in Figure 3, the effects were IMD-pathway–specific since Toll-dependent activation of *drosomycin* by gram-positive or gram-negative bacteria *(M. luteus, E. faecalis, S. aureus, E. cloacae, E. coli, B. subtilis)* were similar in *UAS iPGRP-SC* and *DaGal4;UAS iPGRP-SC* flies. This difference between the role of PGRP-SC1/2 on Toll- and IMD-pathway activation could reflect functional redundancy between amidases for Lys-type PGN which might not exist for m-DAP-type PGN-cleaving enzyme. Alternatively, this could pertain to the difference in the mode of activation of the transmembrane receptors upstream of each pathway.

### PGRP-SC1/2 Function in Larval Immune Response

**Figure 4:**
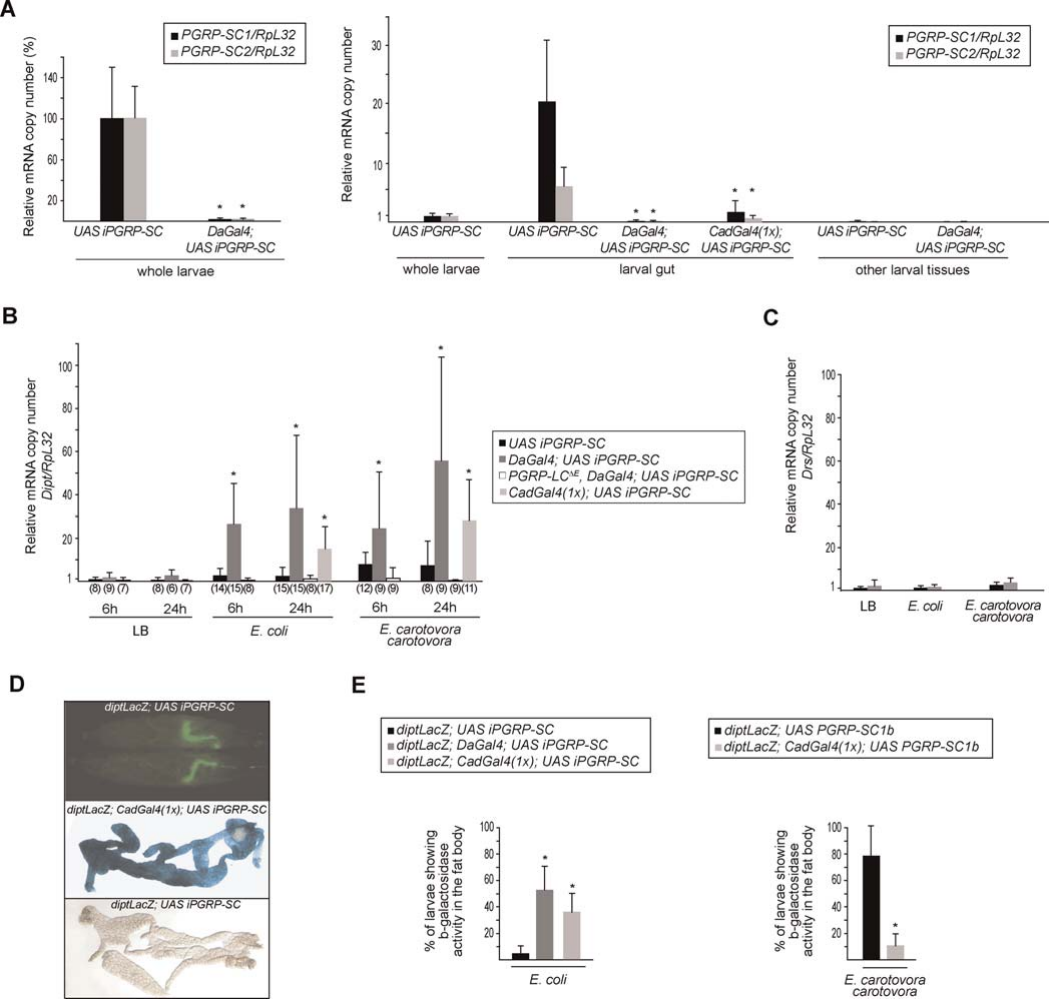
Reduction of PGRP-SC1/2 Levels in the Larval Gut Increases IMD-Pathway Activation after Natural Infection (A) *PGRP-SC1* and *PGRP-SC2* mRNAs *(PGRP-SC/RpL32)* are mainly expressed in the larval gut and are severely reduced in *DaGal4;UAS iPGRP-SC* and *CadGal4;UAS iPGRP-SC* larvae. Each histogram corresponds to the mean value of four independent experiments (± standard deviation). (B) *Diptericin* mrna induction levels *(Dipt/RpL32),* measured 6 h and 24 h after natural infection. Each histogram corresponds to the mean value of variable numbers (shown in parentheses) of independent experiments (± standard deviation). Asterisks indicate that the difference between *DaGal4;UAS iPGRP-SC* or *CadGal4;UAS iPGRP-SC* and control *UAS iPGRP-SC* values is statistically significant (*p <* 0.05). (C) *Drosomycin* mrna induction levels *(Drs/RpL32),* measured 24 h after natural infection. Each histogram corresponds to the mean value of four independent experiments (± standard deviation). (D) Three hours after natural infection with E. coli GFP, bacteria were found to be highly concentrated in the anterior half of the larval gut. In larvae with reduced gut *PGRP-SC* levels *(CadGal4;UAS iPGRP-SC),* feeding on *E.coli* is sufficient to trigger IMD-pathway activation in the fat body after 24 h (visualized here by the use of a *diptericin-LacZ* transgene). (E) Percentage of larvae showing β-galactosidase activity in the fat body 24 h after natural infection. For each genotype, ten larvae were dissected and stained. Each histogram corresponds to the mean value of five independent experiments (± standard deviation). Asterisks indicate that the difference between *diptLacZ;UAS iPGRP-SC* and *diptLacZ;DaGal4;UAS iPGRP-SC* or *diptLacZ;CadGal4;UAS iPGRP-SC* values and between *diptLacZ,UAS iPGRP-SC* and *diptLacZ;CadGal4;UAS iPGRP-SC* values is statistically significant (*p* < 0.05).

We next addressed the role of PGRP-SC proteins in larvae. Previous qualitative analyses indicated that *PGRP-SC1* and *PGRP-SC2* genes are transcribed in almost identical patterns and mostly in the gut cells [25]. Using quantitative RT-PCR, we confirmed that the larval gut is strongly enriched in *PGRP-SC1* and *PGRP-SC2* mRNA and represents the main site of PGRP-SC amidase synthesis at this developmental stage (Figure 4A). In a previously established model of infection by ingestion [31], it was observed that larvae fed with the gram-negative bacteria *Erwinia carotovora carotovora* induce *diptericin* transcription in the fat body. Surprisingly, most of the other gram-negative bacterial species tested in this assay failed to do so. We reasoned that the gut PGRP-SC amidases might act to reduce the PGN immunogenic potential of these bacteria, preventing them from activating a systemic immune response.

To test this hypothesis, *UAS iPGRP-SC* control larvae and *DaGal4;UAS iPGRP-SC* larvae, which exhibit a strong reduction of *PGRP-SC1* and *PGRP-SC2* mRNA levels in their gut (Figure 4A), were fed with various bacterial species. We then monitored *diptericin* expression in whole larvae or, more specifically, in the fat body. As previously reported [31], we found that ingested *E. carotovora carotovora,* but not *E. coli,* was able to activate the IMD pathway in *UAS iPGRP-SC* control larvae (Figure 4B). Strikingly, reducing the PGRP-SC levels induced a strong increase in the expression level of the *diptericin* mRNA at 6 and 24 h after feeding on *E. carotovora carotovora,* as compared to controls (Figure 4B). Under these conditions, E. coli now became a good inducer of *diptericin* expression. This increase in the level of *diptericin* transcription was totally blocked in a *PGRP-LC* mutant background, demonstrating that, in this process, the PGRP-SC amidases act upstream of the IMD-pathway transmembrane receptor (Figure 4B). As shown above for the immune response in adults, reducing the PGRP-SC levels had no effect on Toll-pathway activation in larvae (Figure 4C).

### PGRP-SC1/2 Are Required in the Larval Gut to Dampen the Immune Response

We tested whether similar results could be obtained by reducing PGRP-SC levels specifically in the larval gut using a tissue-specific driver *(CadGal4)* [32]. Consistent with previous reports indicating that *CadGal4* is not a very strong driver[32], we noted that the reduction of *PGRP SC1/2* mRNA levels in the gut were not as pronounced in *CadGal4;UAS iPGRP-SC* than in *DaGal4;UAS iPGRP-SC* larvae (Figure 4A). However, this reduction was sufficient to trigger an activation of the IMD pathway after feeding on E. coli (Figure 4B). Using a *DiptlacZ* reporter transgene, we could show that up to 40% of the *CadGal4;UAS iPGRP-SC* larvae activated the IMD pathway in the fat body after feeding on E. coli (Figure 4D and E). In wild-type control larvae, this percentage was only 5% (Figure 4E). In parallel experiments, we tested the effects of over-expressing the PGRP-SC1b protein in the larval gut. Whereas 80% of the *diptLacZ;UAS PGRP-SC1b* control larvae fed with *E. carotorova carotorova* activated *diptericin* transcription in the fat body, this percentage dropped to 10% in larvae which specifically over-expressed PGRP-SC1b in the gut (Figure 4E). Altogether, these results are compatible with the hypothesis that an essential role of gut PGRP-SC1/2 amidases in larvae is to modulate activation of the IMD pathway. We propose that this modulation is achieved by lowering the amount of immunogenic PGN, most probably via an amidase-dependent degradation. However, we cannot rule out that it is also partly due to sequestration of the PGN.

### Larvae with Reduced PGRP-SC1/2 Levels Are Highly Susceptible to Infection

**Figure 5:**
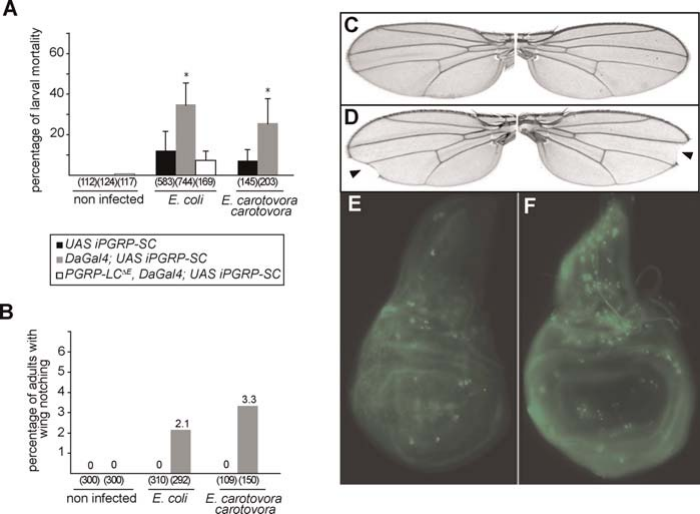
Reduction of PGRP-SC1/2 Levels Sensitizes Larvae to Bacterial Infection (A) The percentage of dead larvae is measured 24 h after natural infection. Numbers in parentheses correspond to the total number of infected larvae. Each histogram corresponds to the mean value of six independent experiments (± standard deviation) for E. coli and five for *E. carotovora carotovora.* Asterisks indicate that the difference between *UAS iPGRP-SC* and *DaGal4;UAS iPGRP-SC* values is statistically significant (*p* < 0.05). (B) The percentage of adults showing wing notching is measured 7 d after natural infection. Numbers in parentheses correspond to the total number of infected larvae. (C–F) Natural infection with *E. carotovora carotorova* and E. coli triggers increased cell death and developmental defects in *DaGal4;UAS iPGRP-SC* flies. Wing imaginal discs dissected from *DaGal4;UAS iPGRP-SC* larvae (F) show higher levels of cell death after natural infection than discs from infected *UAS iPGRP-SC* control larvae (E). Consistently, some *DaGal4;UAS iPGRP-SC* adults derived from infected larvae exhibit wing notching (indicated by arrowheads) (D), which was never observed in infected controls (C).

To evaluate the consequences of the reduction of this immuno-modulatory function, we followed the fate of naturally infected wild-type and PGRP-SC–depleted larvae. We observed that mortality was three to four times higher in depleted larvae fed with E. coli or *E. carotovora carotovora* than in controls (Figure 5A). This increase in larval lethality was totally suppressed in a *PGRP-LC* mutant background, demonstrating that over-activation of the IMD pathway was indeed the cause of larval death. Interestingly, a small percentage of the *DaGal4;UAS iPGRP-SC* larvae that pupariated and eclosed as pharate adults presented developmental defects such as wing notching (Figure 5B–5D). These phenotypes were never observed in control larvae fed with bacteria. To test whether this wing phenotype could be due to increased cell death during larval development, imaginal discs from infected larvae were stained with acridine orange. Wing discs from PGRP-SC–depleted, bacteria-fed larvae showed higher levels of cell death than wing discs from control larvae fed with normal levels of PGRP-SC (Figure 5E and 5F).

## Discussion

The need for a tight balance between initiation and resolution in the control of inflammation in vertebrates has been documented for a long time. Recent reports have reviewed the molecular mechanisms that are put in place to dampen inflammation and to prevent damaging effects associated with a prolonged immune response [33,34]. The data presented here suggest that immune response needs to be tightly regulated also in invertebrates.

Taken together, our data provide novel insights into the physiological roles of PGRPs in *Drosophila.* They show that in addition to the function as a pattern-recognition receptor of some PGRP family members, others can specifically control the level of activation of the IMD signaling pathway. Flies deficient for PGRP-SC1a, PGRP-SC1b, and PGRP-SC2 present a specific over-activation of the IMD pathway. A recent report described an effect of a *PGRP-SC1* mutant *(picky)* on Toll-pathway activation [35], a phenotype that we did not observe in PGRP-SC1/2–depleted flies (see Figure 3). This discrepancy is not yet fully understood but could be explained by the fact that *picky* flies are mutant only for PGRP-SC1a and PGRP-SC1b, whereas PGRP-SC2 is also affected in our PGRP-SC–depleted flies.

Our results indicate that the gut is the main tissue in which the regulation by PGRP-SC proteins is taking place. However, the fact that IMD-pathway over-activation was also detected when bacteria were introduced directly into the circulating hemolymph suggests that these secreted proteins could also be present in the blood or in the circulating hemocytes. We further show that in the absence of a control of the immune response, infection can lead to developmental defects or death by over-activation of the immune pathway. Interestingly, recent reports indicate that other immune-induced pathways can have a harmful effect on fly survival. Salmonella typhimurium–infected flies produce a tumor necrosis factor (TNF)–like cytokine which has been shown to be damaging for the host [36]. In addition, flies in which the gut catalase level is experimentally reduced show high mortality rates after ingestion of microbe-contaminated foods. This has been interpreted as evidence that infection-mediated induction of reactive oxygen species (such as H_2_O_2_) must be tightly balanced to avoid larval lethality [32]. In this respect, our data indicate that PGRP-SC1/2 may act as detoxifying proteins for bacterial PGN in flies. Although we did not demonstrate that the amidase function of these PGRPs is required for this effect, in vitro biochemical data strongly suggest that it is the case. Similar functions have recently been attributed to enzymes which reduce the immunogenic potential of lipopolysaccharide during vertebrate immune response [37].

The results presented here are consistent with previous data showing that over-expression of some components of the IMD pathway are larval lethal. However, the molecular mechanisms by which over-activation of the IMD pathway leads to lethality remain unknown. A number of observations may provide clues about this issue: (i) several components of the IMD pathway are homologous to mammalian proteins involved in signaling through the TNF receptor, a pathway known to trigger apoptosis [38]; and (ii) the MAP3 kinase TAK1, which is an essential component of the IMD pathway, has been shown to function both as an IκB kinase (regulating *diptericin* expression) and as a JNK (c-Jun *N*-terminal kinase) kinase [39]. It is significant in this context that inappropriate activation of the JNK signaling cascade in the wing disc leads to apoptotic pathway-dependent morphological defects [40]. Further investigations will be needed to clarify the molecular links existing between the activation of the *Drosophila* IMD pathway and the developmental defects which we observed; in particular, a role of the apoptosis pathways in this process should be considered. Finally, it will be of interest to investigate whether amidases or other PGN-modifying enzymes are involved in modulating bacteria-induced immune response in mammals. In this respect, it is intriguing that one human PGRP family member (PGRP-Iβ) is expressed in the esophagus [21], which evokes the gut expression of PGRP-SC.

## Materials and Methods

### Bacterial strains.

The following microorganisms were used: *E. coli, M. luteus, E. carotovora carotovora 15, B. subtilis, E. cloacae, E. faecalis, S. aureus,* and *E. coli AmpR.*

### *Drosophila* strains.

*IKKγ^key1^* is a loss-of-function mutation allele. *PGRP-LC^Δ^* ^E^ is a complete deletion of the *PGRP-LC* locus. Flies carrying either of these mutations are unable to activate the IMD pathway*. PGRP-SA^seml^* is a point-mutation null allele which prevents Toll-pathway activation by some bacteria. All these alleles have been previously described [8]. Daughterless Gal4 *(DaGal4)* and Caudal Gal4 *(CadGal4)* are transgenic strains in which all *(DaGal4)* or only the gut *(CadGal4)* cells express the yeast Gal4 transcription factor. In *HsGal4* flies, the production of the Gal4 protein is inducible by a heat pulse. *DaGal4;UAS iPGRP-SC* are strains in which all the cells produced *PGRP-SC* dsRNA targeting the endogenous *PGRP-SC* transcript to degradation by RNA interference. *Drosophila* strains produced include: *PGRP-SA, PGRP-SC1a, PGRP-SC1b, PGRP-SC2, PGRP-SD, PGRP-LB, PGRP-LC, PGRP-LE, IKKγ,* and *RpL32.*

### Septic injuries, bacterial growth, and fly survival experiments.

Cells from overnight bacterial cultures were recovered by centrifugation at 3,000 *g* for 10 min at room temperature. The supernatant was discarded and the pellet was resuspended in fresh Luria-Bertani (LB) media. Cell suspensions were serially diluted in PBS, and the concentration of cells was determined by optical-density measurement. Flies were anaesthetized with CO_2_ and were infected by pricking the dorsal thorax with a thin tungsten needle that had previously been dipped into cultures of the appropriate bacterial strains*.*

In bacterial-growth experiments, five 4-d-old adults were infected with *E. coli* or with *E. cloacae.* Twenty-four hours later, flies were homogenized in 400 μl of LB media, and various dilutions were spread on LB plates containing ampicillin for E. coli (50 μg/ml) or on LB plates for *E. cloacae.* The number of colony-forming units per fly was determined through overnight growth on plates. For survival experiments, 25 flies of each tested genotype were pricked and transferred each day into new vials with fresh medium. Survival assays were repeated four times.

### Generation of *UAS iPGRP-SC, UAS iPGRP-SA,* and *UAS PGRP-SC1b* constructs.

The *UAS iPGRP-SC* plasmid was constructed by inserting a 558-bp PCR fragment corresponding to the *PGRP-SC1a* coding sequence flanked by *BamH*I and *Nhe*I sites between the *Nhe*I–*BamH*I sites (sense) and the *Xba*I–*Bgl*II sites (antisense), respectively, into the RNAi vector [41]; primers were as follows: forward 5′-GGGGGATCCATGGTTTCCAAAGTGGCTCTC-3′, reverse 5′-GGGGCTAGCCTAGCCAGACCAGTGGGA CCA-3′. The same strategy was used for plasmid *UAS iPGRP-SA* by inserting a 612-bp fragment corresponding to the *PGRP-SA* coding sequence; primers were as follows: forward 5′- GGGGGATCCATGCAGCCGGTTCGATTCGGA-3′, reverse 5′- GGGG CTAGCTCCGA TGGAAGTTTATCCACA-3′. For plasmid *UAS PGRP-SC1b,* the wild-type *PGRP-SC1b* cDNA was obtained by PCR using the EST GH07464 (FlyBase, http://flybase.bio.indiana.edu) as a template. This fragment was then sub-cloned into the pUAST vector. Primers were as follows: forward 5′-GGGGGAATTCATGGTTTCCAAAGTGGCTCT-3′, reverse 5′-GGGGTCTAGACACTCTAACCAGACCAGTGG-3′. After sequencing, the constructs were injected into *w^1118^* embryos.

### Natural infection of larvae.

Wandering third-instar larvae were placed in a tube containing a mixture of a concentrated overnight bacterial culture with 5 % sucrose, and were incubated for 30 min at 25 °C. For controls, larvae were incubated in LB broth supplemented by 5% sucrose. Larvae were then transferred to grape-juice plates and incubated at 25 °C. Larvae were monitored for *diptericin* transcription by real-time Q-PCR 6 and 24 h after infection. For observation of developmental defects, larvae were transferred to standard cornmeal-agar medium and incubated at 25 °C until hatching.

### Quantitative real-time PCR.

Real-time Q-PCR was performed as described [8]. Primers were as follows: *PGRP-SC1,* forward 5′-aagcgatcgtcaactattacagc-3′, reverse 5′-gagagccactttggaaacCA-3′; *PGRP-SC1b* (for over-expression checking), forward 5′-AGCTTCCTGGGCAACTACAA-3′, reverse 5′-GAGATCATGTTCGGCTCCAG-3′; *PGRP-SC2,* forward 5′-TGACCATCATCTCCAAGTCG-3′, reverse 5′-CAGCGAGGTCTTGCTCGT-3′; *PGRP-SA,* forward 5′-GCTTCGTTGGGACTCCACTA-3′; reverse 5′-CGTGTGATGGATG ACCACAT-3′. *RpL32, diptericin, drosomycin,* and *PGRP-SD* primers are described in [8,11].

### Acridine orange staining.

Imaginal discs were dissected in PBS, incubated in a 5 μg/ml AO solution for 1 min, rinsed three times for 5 min in fresh PBS, and mounted in glycerol.

## Supporting Information

Figure S1 Schematic Representation of the PGN Structure (578 KB PDF)

Figure S2 PGRP-SC Alignments and Phylogeny (1.4 MB PDF)

### Accession Numbers

The FlyBase (http://flybase.bio.indiana.edu) accession numbers for the *Drosophila* strains produced include *IKKγ* (CG16910), *PGRP-LB* (CG14704), *PGRP-LC* (CG4432), *PGRP-LE* (CG8995), *PGRP-SA* (CG11709), *PGRP-SC1a* (CG14746), *PGRP-SC1b* (CG8577), *PGRP-SC2* (CG14745), *PGRP-SD* (CG7496), and *RpL32* (CG7939).

The Swiss-Prot Enzyme Nomenclature database (http://www.expasy.org/enzyme) accession number for NAMLAA is EC3.5.1.28.

## Abbreviations

DIF: dorsal-related immune factor
IMD: immune deficiency
JNK: c-Jun *N*-terminal kinase
LB: Luria-Bertani
m-DAP: meso-diaminopimelic acid
NAMLAA: *N*-acetylmuramyl-l-alanine amidases
PGN: peptidoglycan
PGRP: peptidoglycan-recognition protein
TNF: tumor necrosis factor
