# Supplementary material for: Downregulation of the Drosophila Immune Response by Peptidoglycan-Recognition Proteins SC1 and SC2
Source: PLoS Pathog. 2006 Feb 24;2(2):e14. doi: 10.1371/journal.ppat.0020014 (PMC1383489; doi:10.1371/journal.ppat.0020014)
Supplement: Figure S1 — (578 KB PDF) [file ppat.0020014.sg001.pdf]

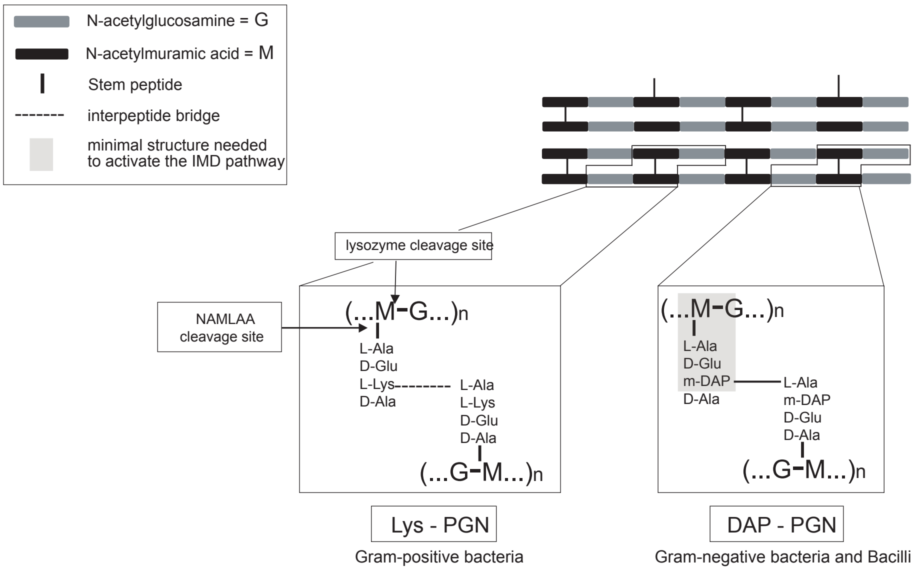

Figure S1.Schematic Representation of the Peptidoglycan Structure.

Peptidoglycan (PGN) is a polymer of alternating N-acetylmuramic acid (M) and N-acetylglucosamine (G) linked by stem peptide bridges. Gram-positive bacteria have a Lysine at the third position of this bridge(Lys-PGN) whereas Gram-negative bacteria and Bacilli have a meso-diaminopimelic acid (m-DAP) at this position (DAP-PGN). The stem peptides of DAP-PGN are generally directly cross-linked whereas those of Lys-PGN are usually cross-linked through an "interpeptide bridge" that varies in length and amino acid composition according to the Gram-positive bacteria species. The minimal structure required to activate the IMD signaling pathway consists in two sugars and the three first amino acids of the stem peptide.
