# Supplementary material for: Downregulation of the Drosophila Immune Response by Peptidoglycan-Recognition Proteins SC1 and SC2
Source: PLoS Pathog. 2006 Feb 24;2(2):e14. doi: 10.1371/journal.ppat.0020014 (PMC1383489; doi:10.1371/journal.ppat.0020014)
Supplement: Figure S2 — (1.4 MB PDF) [file ppat.0020014.sg002.pdf]

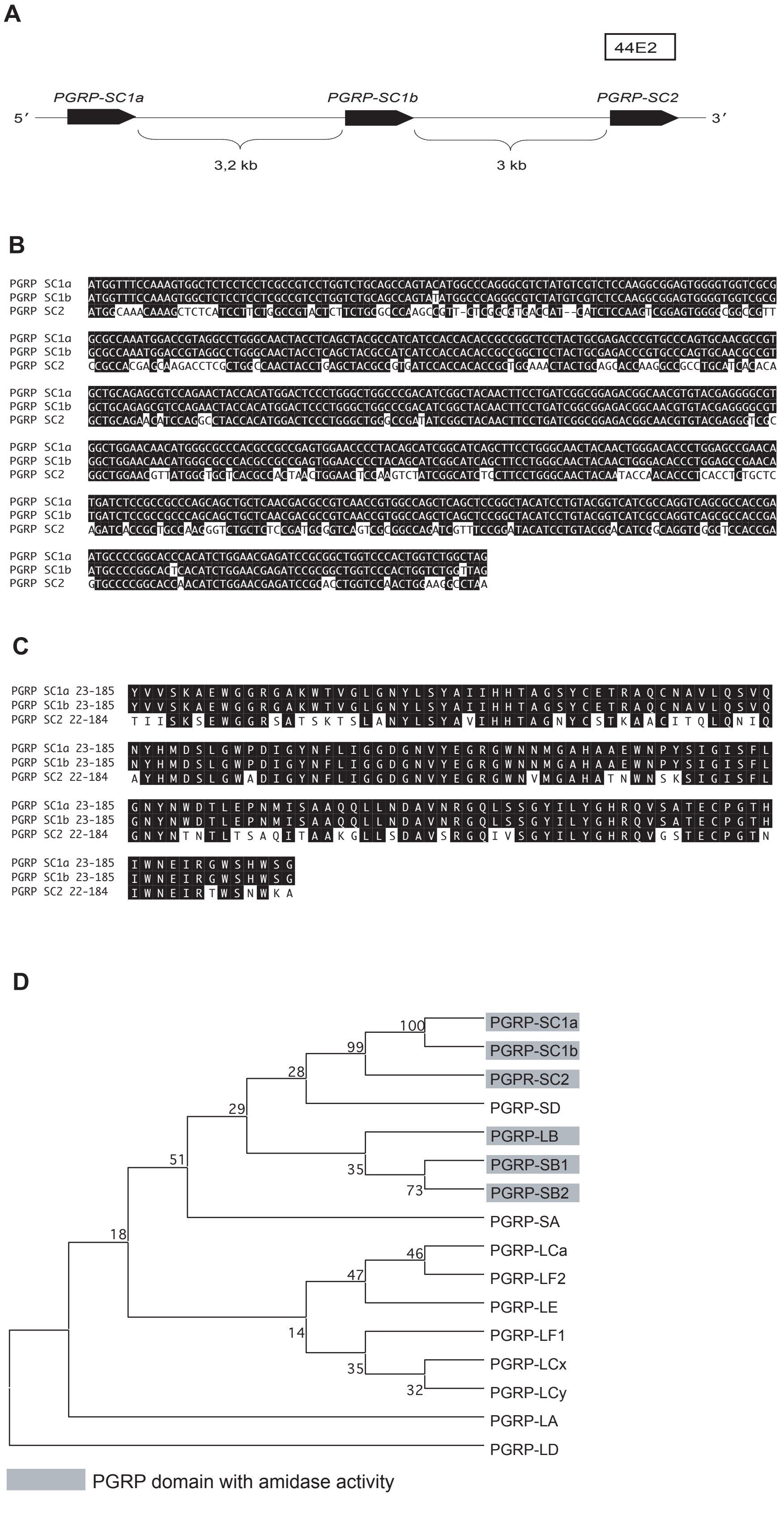

Figure S2 . PGRP-SC Alignments and Phylogeny.
(A) Structure of the PGRP-SC genomic locus.
(B) Nucleotide alignment shows that there is 74% sequence identity between *PGRP-SC1a* and *PGRP-SC2*.
(C) This alignment shows that there is 70,6% amino acid sequence identity between the PGRP domain of PGRP-SC1 and PGRP-SC2.
(D) Phylogenetic tree of PGRP domains established by bootstrap analysis using MEGA3 software. Numbers correspond to the percentage of 500 replications.
